# Supplementary figures and images for: Pattern of tamoxifen-induced Tie2 deletion in endothelial cells in mature blood vessels using endo SCL-Cre-ERT transgenic mice
Source: PLoS One. 2022 Jun 8;17(6):e0268986. doi: 10.1371/journal.pone.0268986 (PMC9176780; doi:10.1371/journal.pone.0268986)

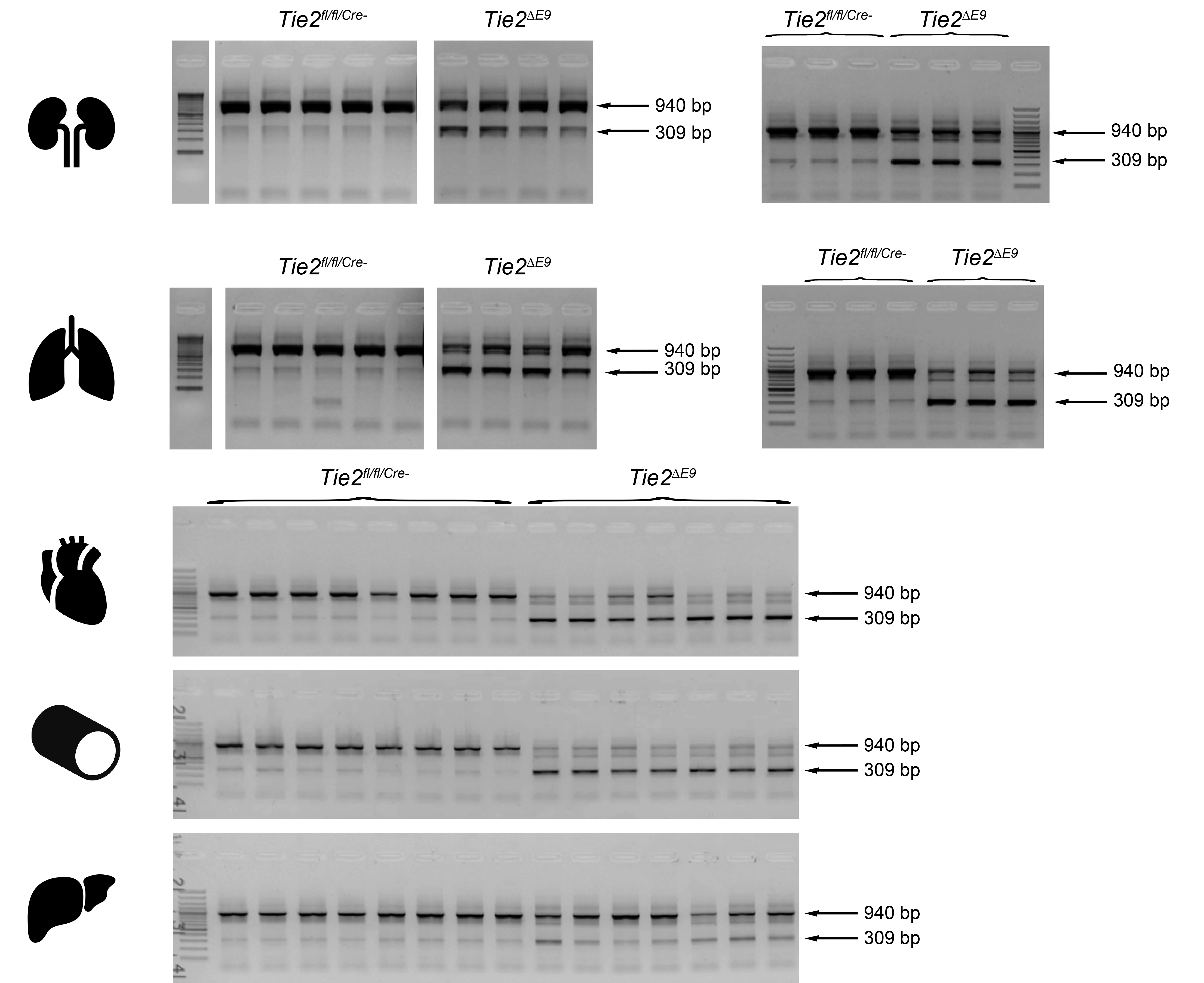

Supplement: S1 Fig — After tamoxifen-induced activation of Cre-recombinase, the presence (a 940 base pair (bp) PCR product) or absence (a 309 bp PCR product) of Tie2 exon 9 was confirmed by genomic PCR in kidney, lung, heart, aorta, and liver tissue of Tie2ΔE9 knockout mice (n = 7) and Tie2fl/fl/Cre- control mice (n = 8). Lanes show genomic PCR products for individual mice. Kidneys and lungs were genotyped in two separate groups of mice, hence PCR products are shown on separate gels. (TIF) [file pone.0268986.s001.tif]

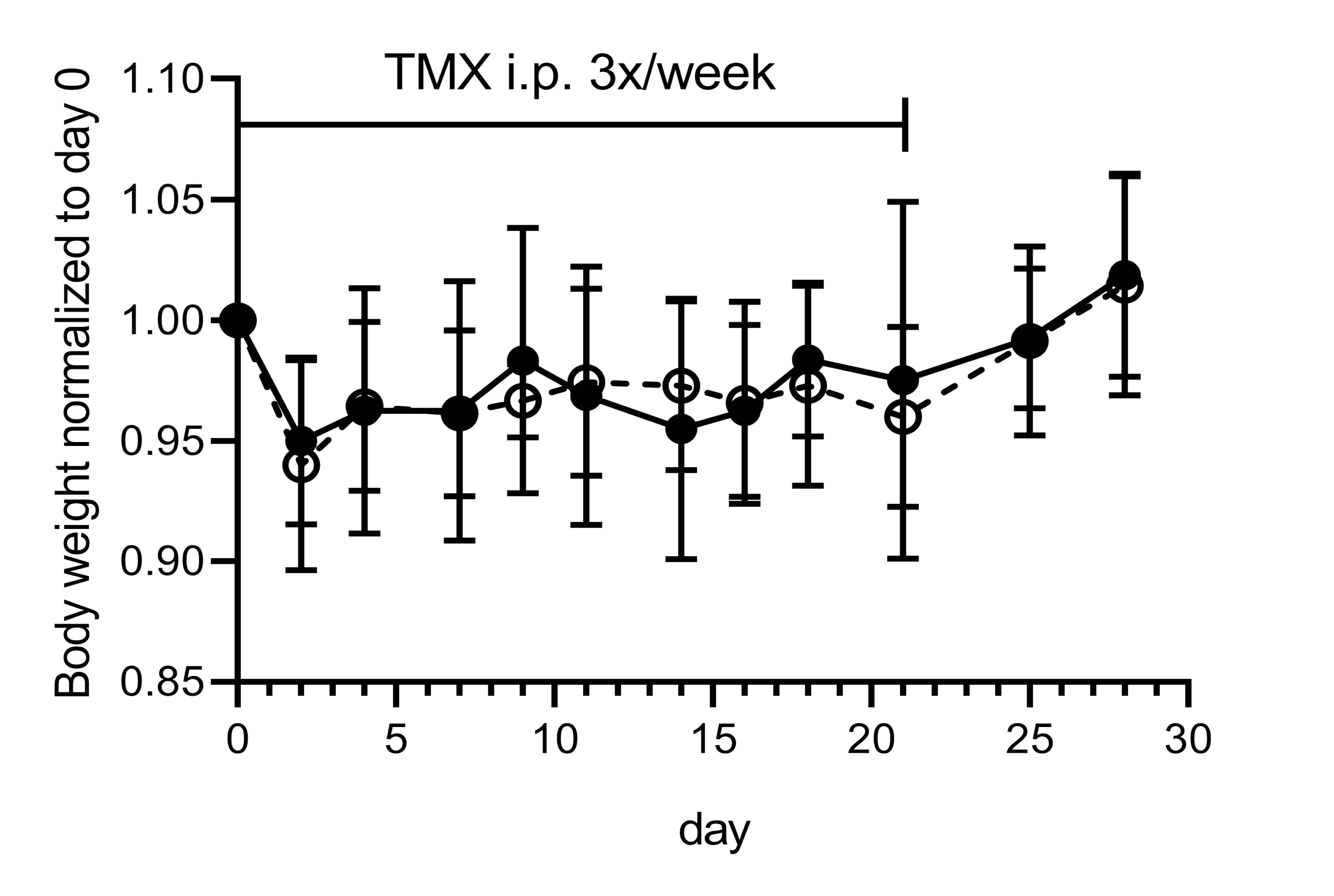

Supplement: S2 Fig — Tie2fl/fl/Cre- control mice and Tie2ΔE9 knockout mice were intraperitoneally injected with tamoxifen (4mg/injection), three times a week for a period of three weeks. Body weights were measured at 2-3-day intervals and normalized to the weights at the start of the experiment. Symbols represent mean values ± SD (error bars). Closed circles with closed line: Tie2fl/fl/Cre- mice (n = 8); open circles with dashed line: Tie2ΔE9 mice (n = 7). (TIF) [file pone.0268986.s002.tif]

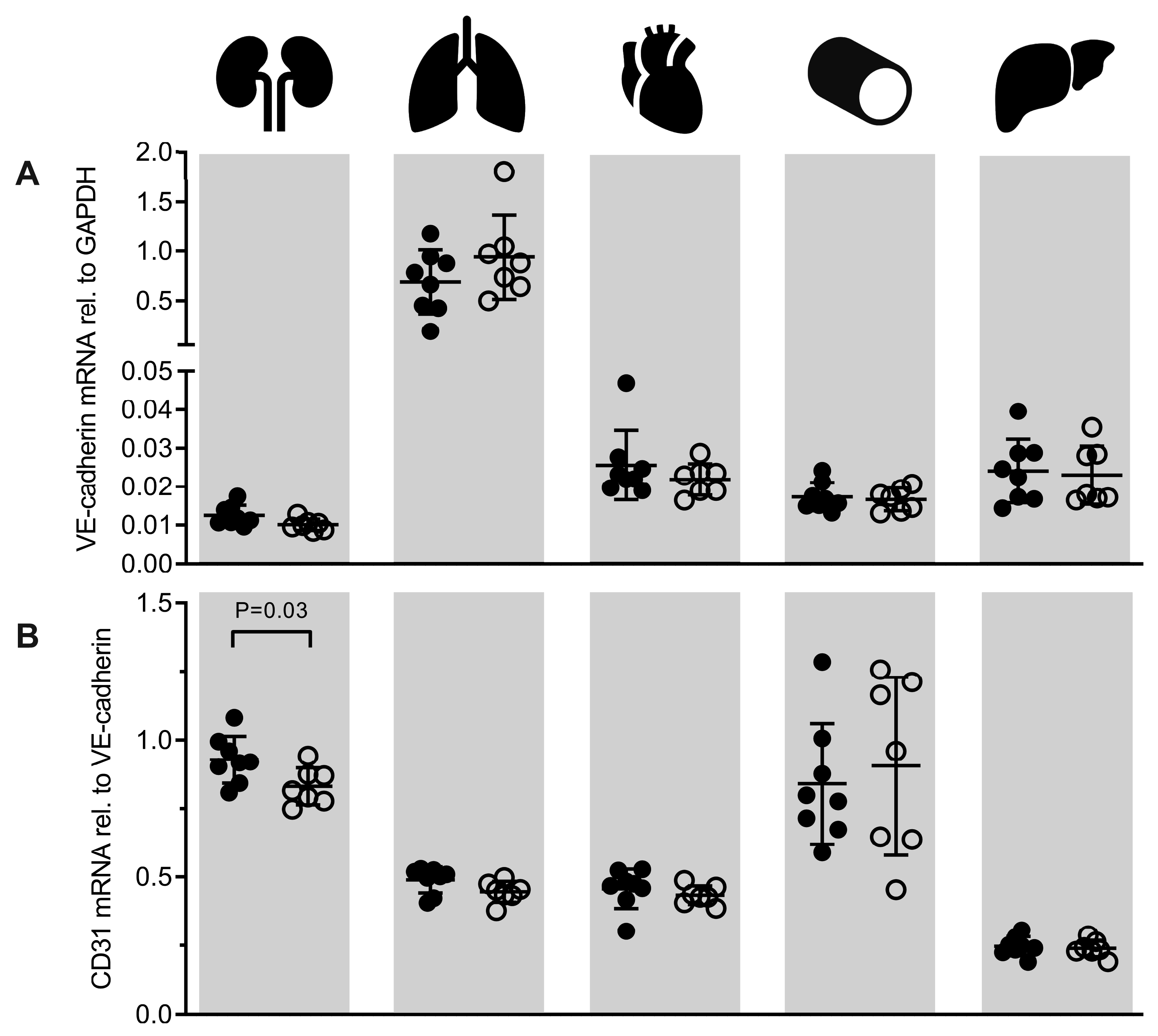

Supplement: S3 Fig — mRNA expression of the pan-endothelial genes VE-cadherin and CD31 was quantified in organs by RT-qPCR. (A) Gene expression of VE-cadherin was normalized to the housekeeping gene Gapdh. (B) Gene expression of CD31 was normalized to VE-cadherin. Graphs show individual values and means (black lines) ± SD (error bars). Closed circles: Tie2fl/fl/Cre- control mice (n = 8); open circles: Tie2ΔE9 knockout mice (n = 7). (TIF) [file pone.0268986.s003.tif]

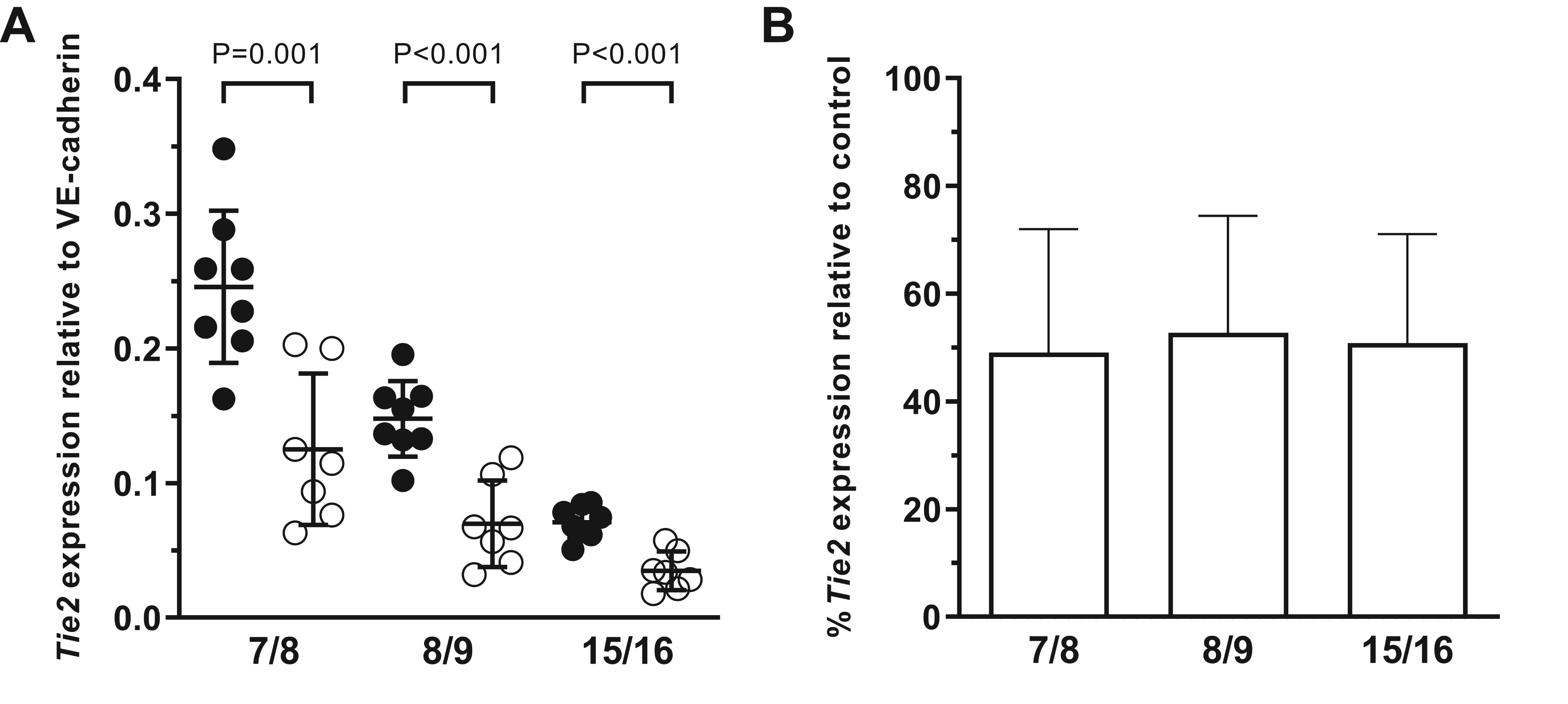

Supplement: S4 Fig — Tie2 mRNA expression levels were determined in lungs of Tie2fl/fl/Cre- control mice and Tie2ΔE9 knockout mice after tamoxifen treatment by RT-qPCR using 3 different primer/probe sets that bind at the boundary of exon 7/8, exon 8/9, or exon 15/16 of Tie2. (A) Tie2 expression in Tie2fl/fl/Cre- control, and Tie2ΔE9 knockout samples. Exon binding sites of the primer/probe sets are indicated under the X-axis. Graph shows individual values and means (black lines) ± SD (error bars). Closed circles: Tie2fl/fl/Cre- mice (n = 8); open circles: Tie2ΔE9 mice (n = 7) (B) Percent knockout of Tie2 mRNA in Tie2ΔE9 knockout samples relative to control mice. Bars shows means ± SD (error bars). (TIF) [file pone.0268986.s004.tif]
