# Supplementary material for: Pattern of tamoxifen-induced Tie2 deletion in endothelial cells in mature blood vessels using endo SCL-Cre-ERT transgenic mice
Source: PLoS One. 2022 Jun 8;17(6):e0268986. doi: 10.1371/journal.pone.0268986 (PMC9176780; doi:10.1371/journal.pone.0268986)
Supplement: S1 Table — Differences in mRNA levels of Tie1, Tie2, Ang1, and Ang2 (Fig 4) between organs of Tie2fl/fl/Cre- control mice were determined by Sidak’s multiple comparisons test. Adjusted P values are reported. (DOCX) [file pone.0268986.s005.docx]

**S4 Table.**

| **Organ** | **Tie1** | **Tie2** | **Ang1** | **Ang2** |
| --- | --- | --- | --- | --- |
| Kidney vs. Lung | <0,0001 | <0,0001 | <0,0001 | <0,0001 |
| Kidney vs. Liver | <0,0001 | <0,0001 | <0,0001 | <0,0001 |
| Kidney vs. Heart | 0,0471 | <0,0001 | <0,0001 | <0,0001 |
| Kidney vs. Aorta | 0,0314 | <0,0001 | <0,0001 | <0,0001 |
| Lung vs. Liver | 0,8963 | 0,1485 | <0,0001 | 0,9915 |
| Lung vs. Heart | <0,0001 | 0,9780 | <0,0001 | >0,9999 |
| Lung vs. Aorta | <0,0001 | 0,6470 | 0,1130 | 0,0010 |
| Liver vs. Heart | <0,0001 | 0,7734 | <0,0001 | 0,9942 |
| Liver vs. Aorta | <0,0001 | 0,9947 | 0,0016 | 0,0003 |
| Heart vs. Aorta | >0,9999 | 0,9990 | <0,0001 | 0,0009 |
