# Supplementary material for: Pattern of tamoxifen-induced Tie2 deletion in endothelial cells in mature blood vessels using endo SCL-Cre-ERT transgenic mice
Source: PLoS One. 2022 Jun 8;17(6):e0268986. doi: 10.1371/journal.pone.0268986 (PMC9176780; doi:10.1371/journal.pone.0268986)
Supplement: S2 Table — Calculated ratios of mRNA expression levels in organs of Tie2fl/fl/Cre- control mice as presented in Fig 4. (DOCX) [file pone.0268986.s006.docx]

**S6 Table.**

| **Organ** | ***Tie2/Tie1* ratio** | ***Ang1/Ang2* ratio** |
| --- | --- | --- |
| Kidney | 1.6 | 1.6 |
| Lung | 1.3 | 9.1 |
| Heart | 0.6 | 16.5 |
| Aorta | 0.5 | 1.1 |
| Liver | 1.1 | 2.9 |
