# Supplementary material for: Pattern of tamoxifen-induced Tie2 deletion in endothelial cells in mature blood vessels using endo SCL-Cre-ERT transgenic mice
Source: PLoS One. 2022 Jun 8;17(6):e0268986. doi: 10.1371/journal.pone.0268986 (PMC9176780; doi:10.1371/journal.pone.0268986)
Supplement: S3 Table — Differences in mRNA level ratios of Tie2/Tie1 and Ang1/Ang2 of Tie2fl/fl/Cre- control mice (S2 Table) were determined by Sidak’s multiple comparisons test. Adjusted P values are reported. (DOCX) [file pone.0268986.s007.docx]

**S7 Table.**

| **Organ** | **Tie2/Tie1** | **Ang1/Ang2** |
| --- | --- | --- |
| Kidney vs. Lung | 0.0033 | <0.0001 |
| Kidney vs. Liver | <0.0001 | 0.6019 |
| Kidney vs. Heart | <0.0001 | <0.0001 |
| Kidney vs. Aorta | <0.0001 | 0.9994 |
| Lung vs. Liver | 0.1954 | <0.0001 |
| Lung vs. Heart | <0.0001 | <0.0001 |
| Lung vs. Aorta | <0.0001 | <0.0001 |
| Liver vs. Heart | <0.0001 | <0.0001 |
| Liver vs. Aorta | <0.0001 | 0.1968 |
| Heart vs. Aorta | >0.9999 | <0.0001 |
