# Supplementary material for: Pattern of tamoxifen-induced Tie2 deletion in endothelial cells in mature blood vessels using endo SCL-Cre-ERT transgenic mice
Source: PLoS One. 2022 Jun 8;17(6):e0268986. doi: 10.1371/journal.pone.0268986 (PMC9176780; doi:10.1371/journal.pone.0268986)
Supplement: S1 Raw images — Each raw image is labeled and annotated to identify corresponding bands used in the manuscript figures by use of Adobe Photoshop v22.1.1. (PDF) [file pone.0268986.s008.pdf]

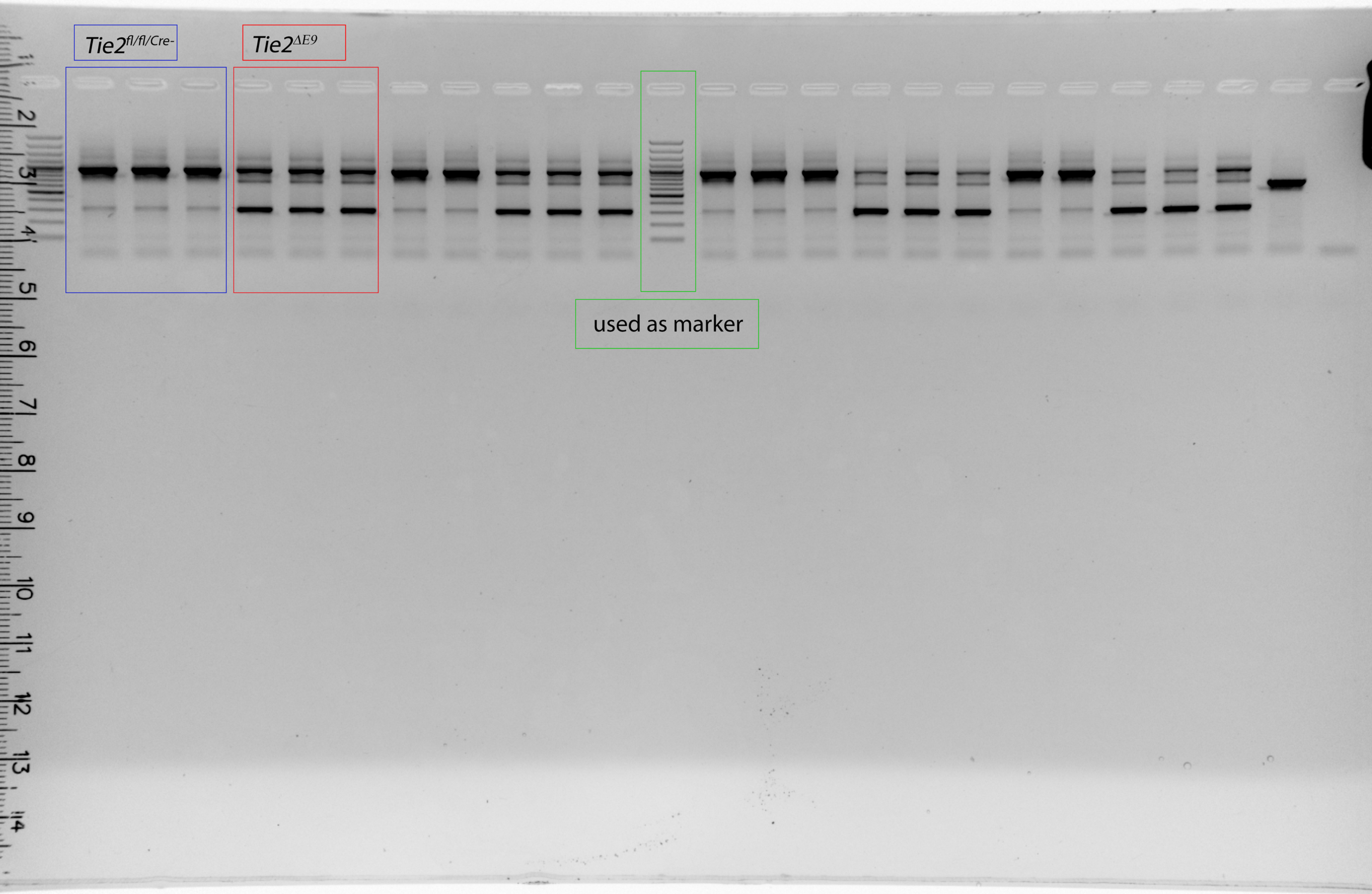

unedited gel for Fig1

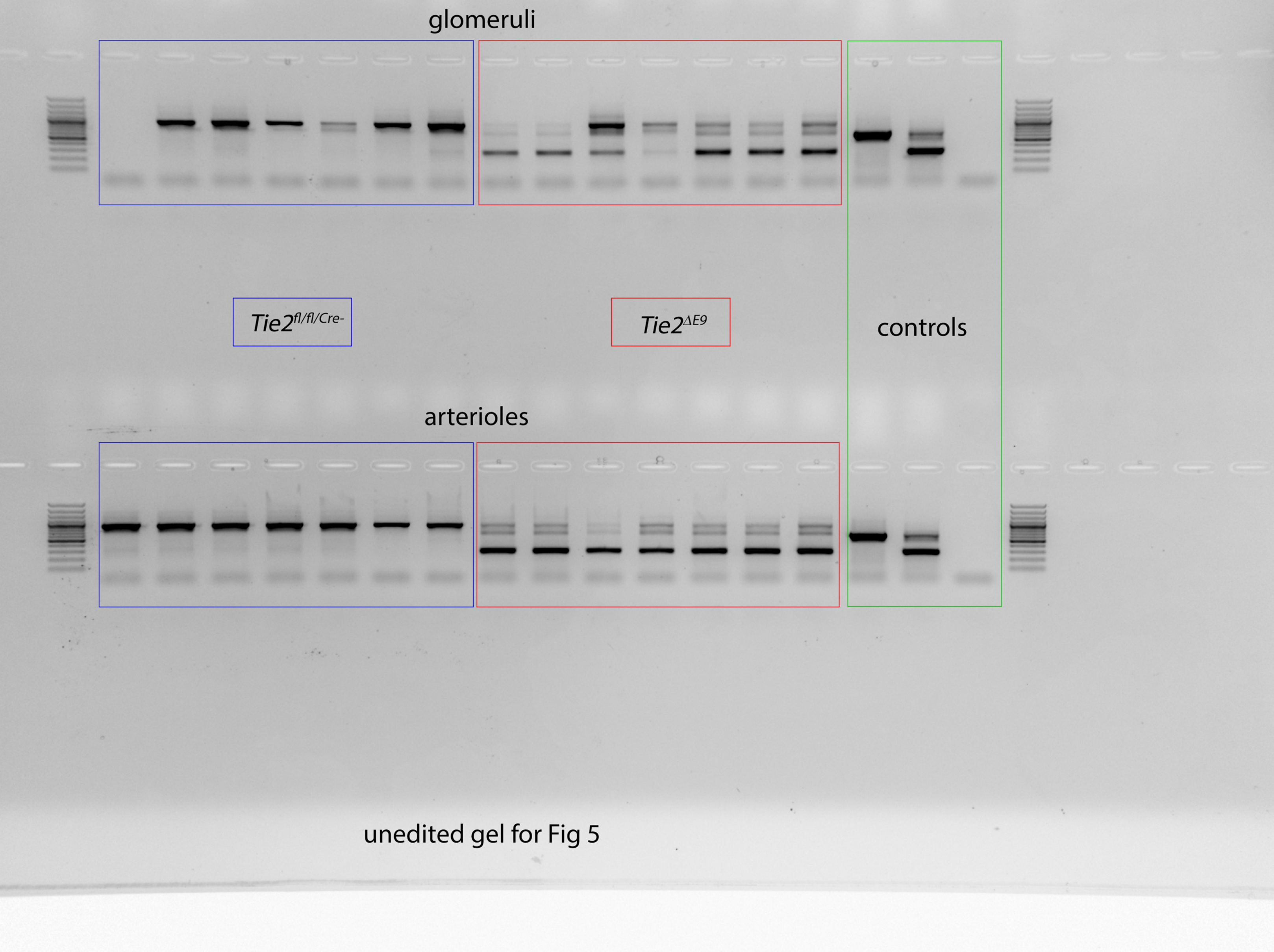

unedited gel for Fig 5

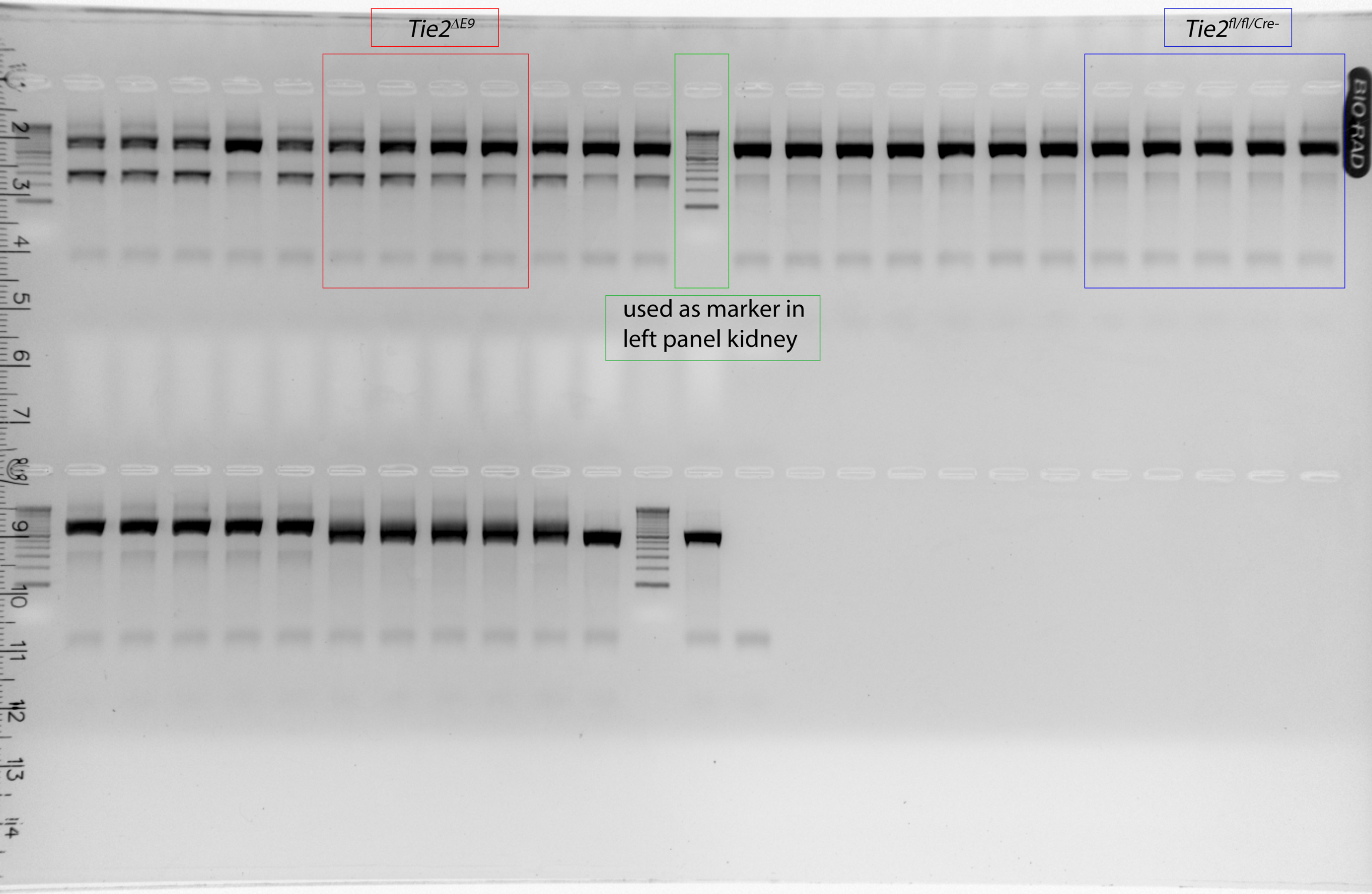

unedited gel S1Fig - kidney

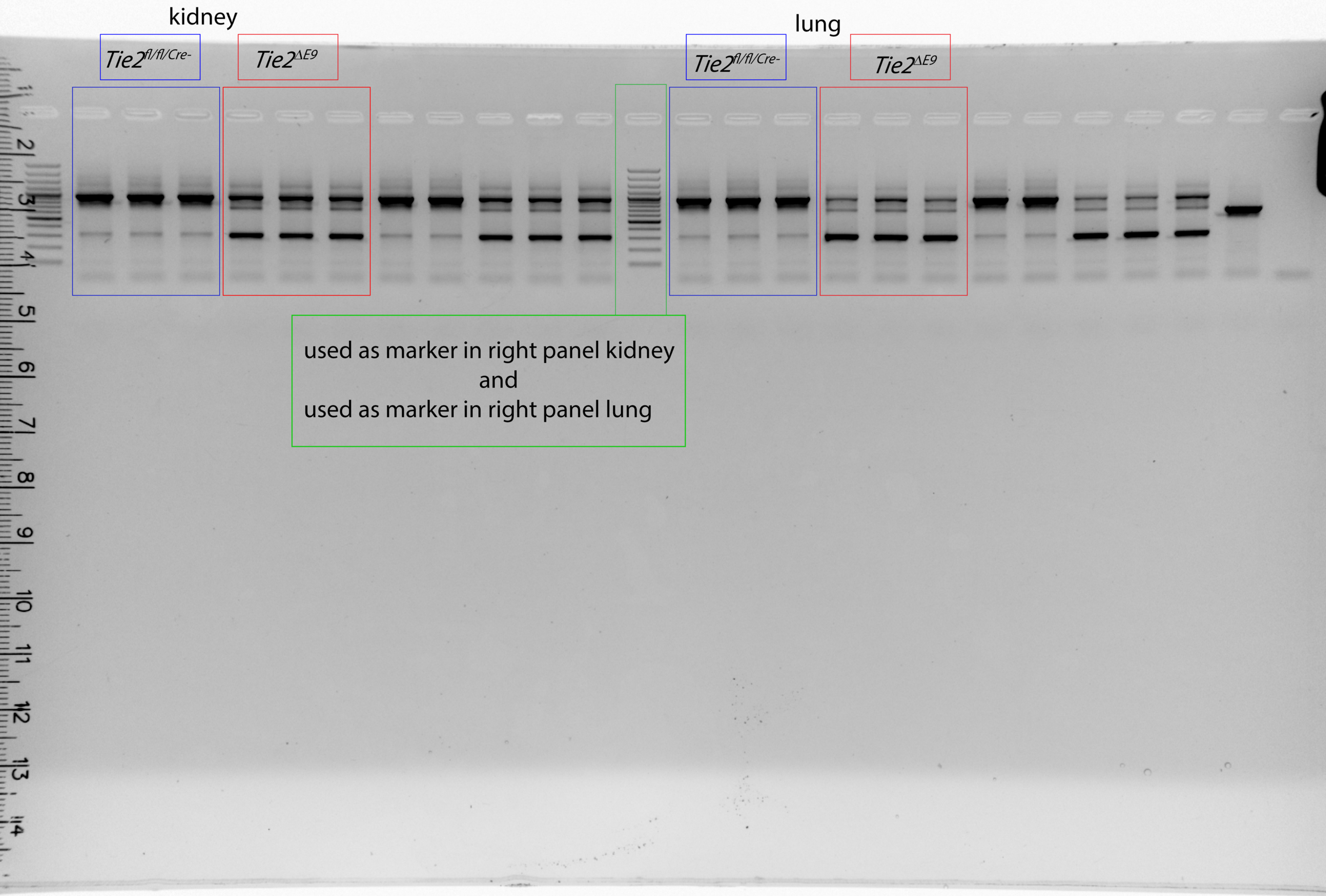

unedited gel S1Fig - kidney and lung

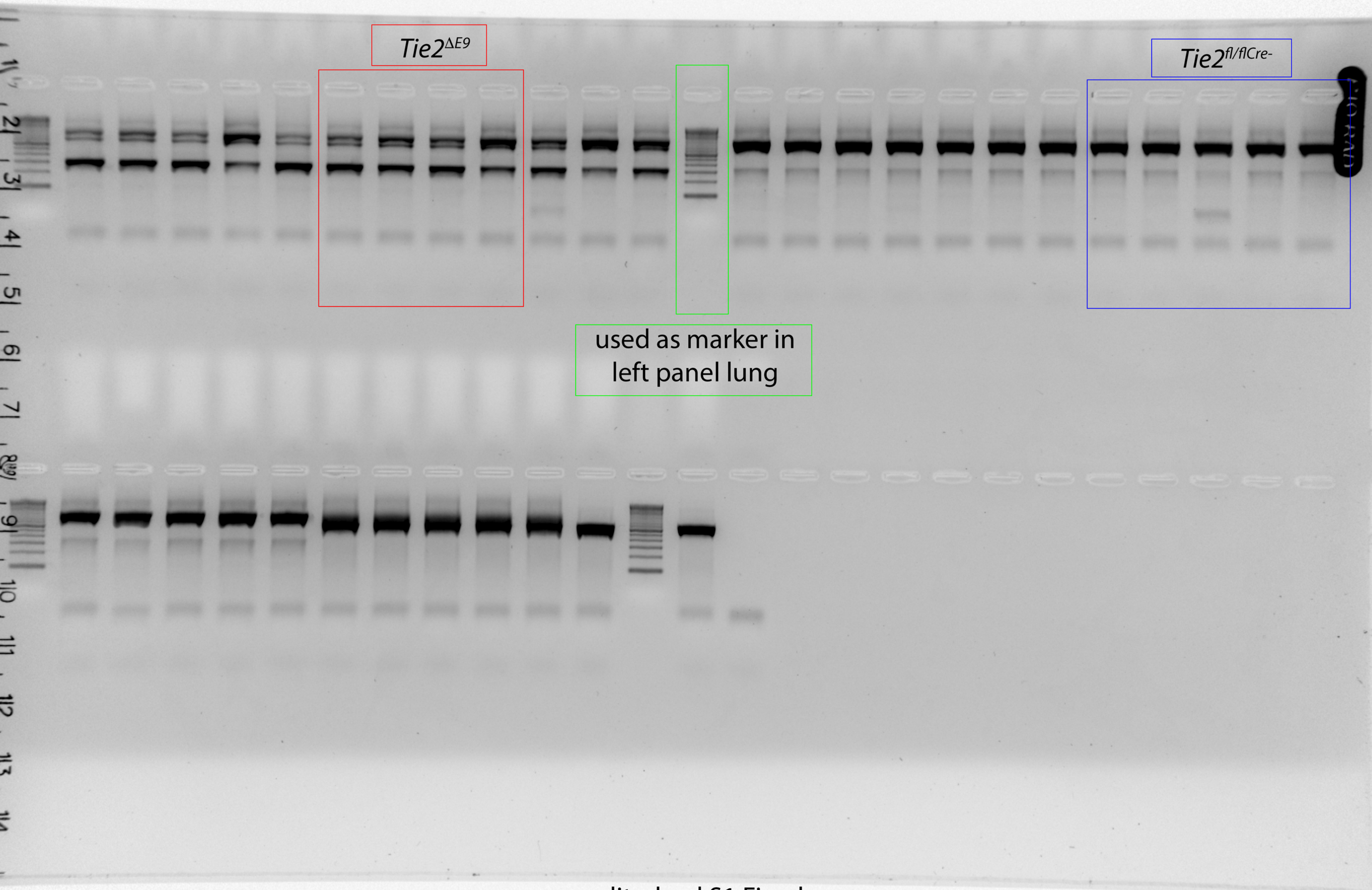

unedited gel S1 Fig - lung

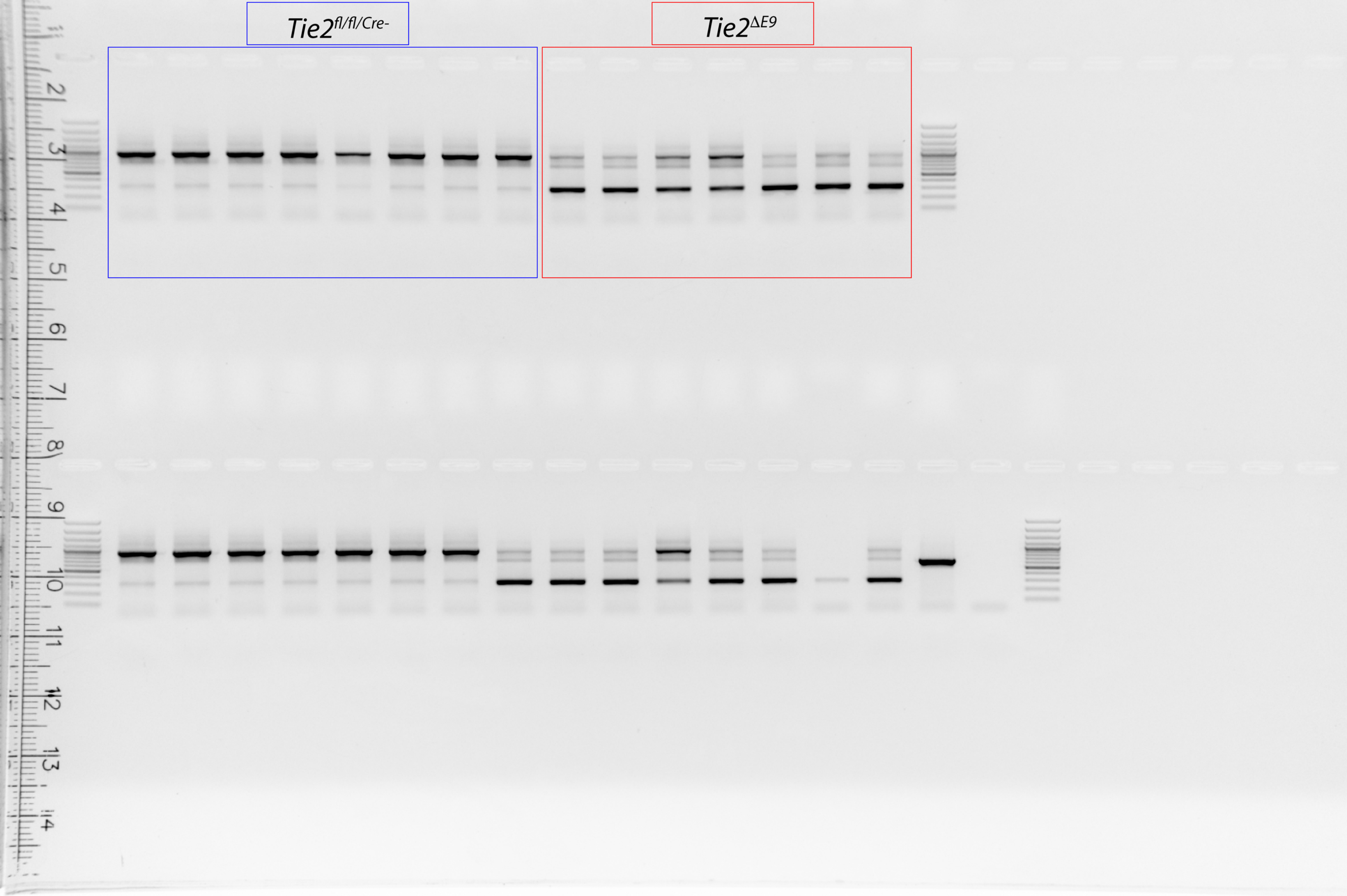

unedited gel S1 Fig - heart

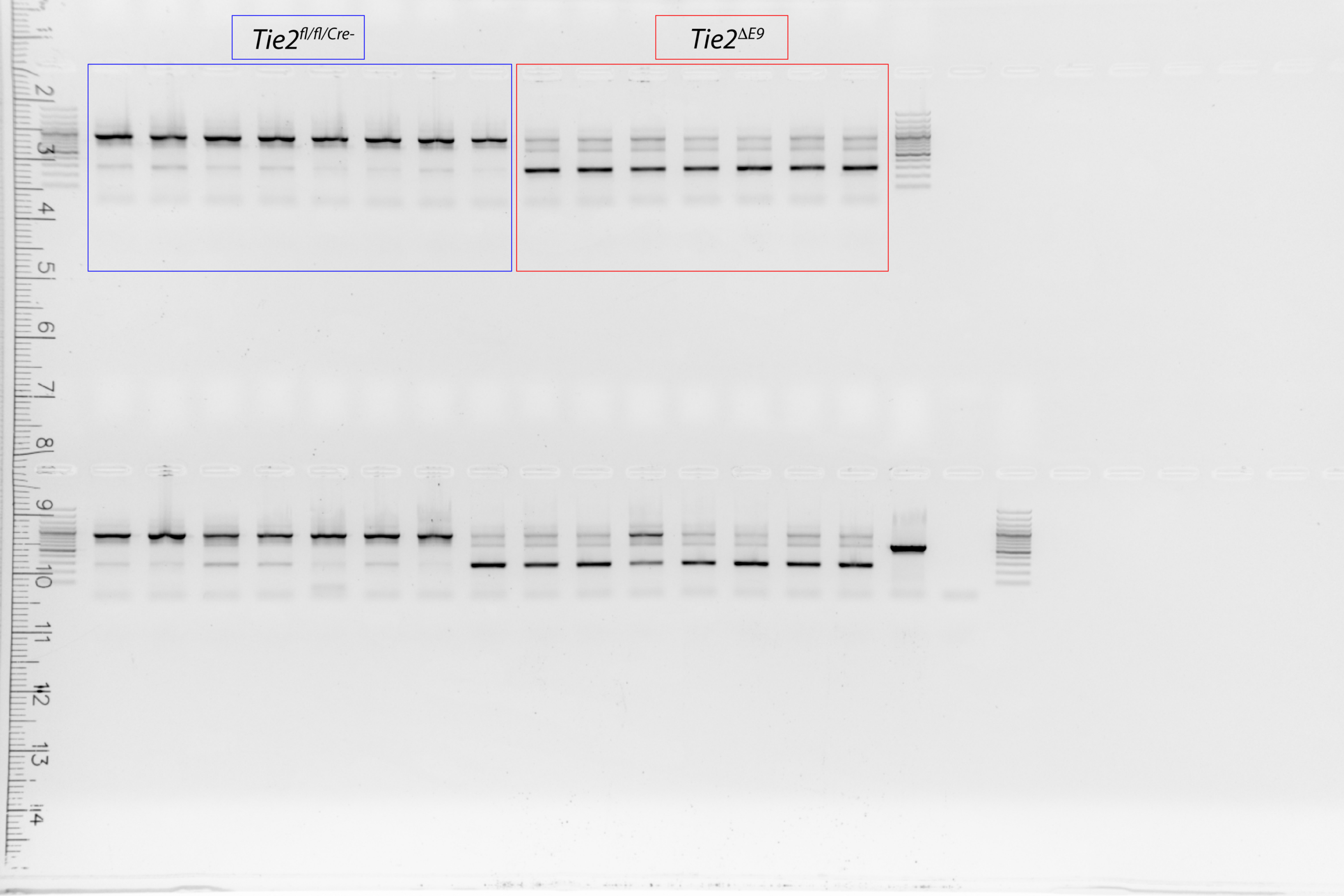

unedited gel S1 Fig - aorta

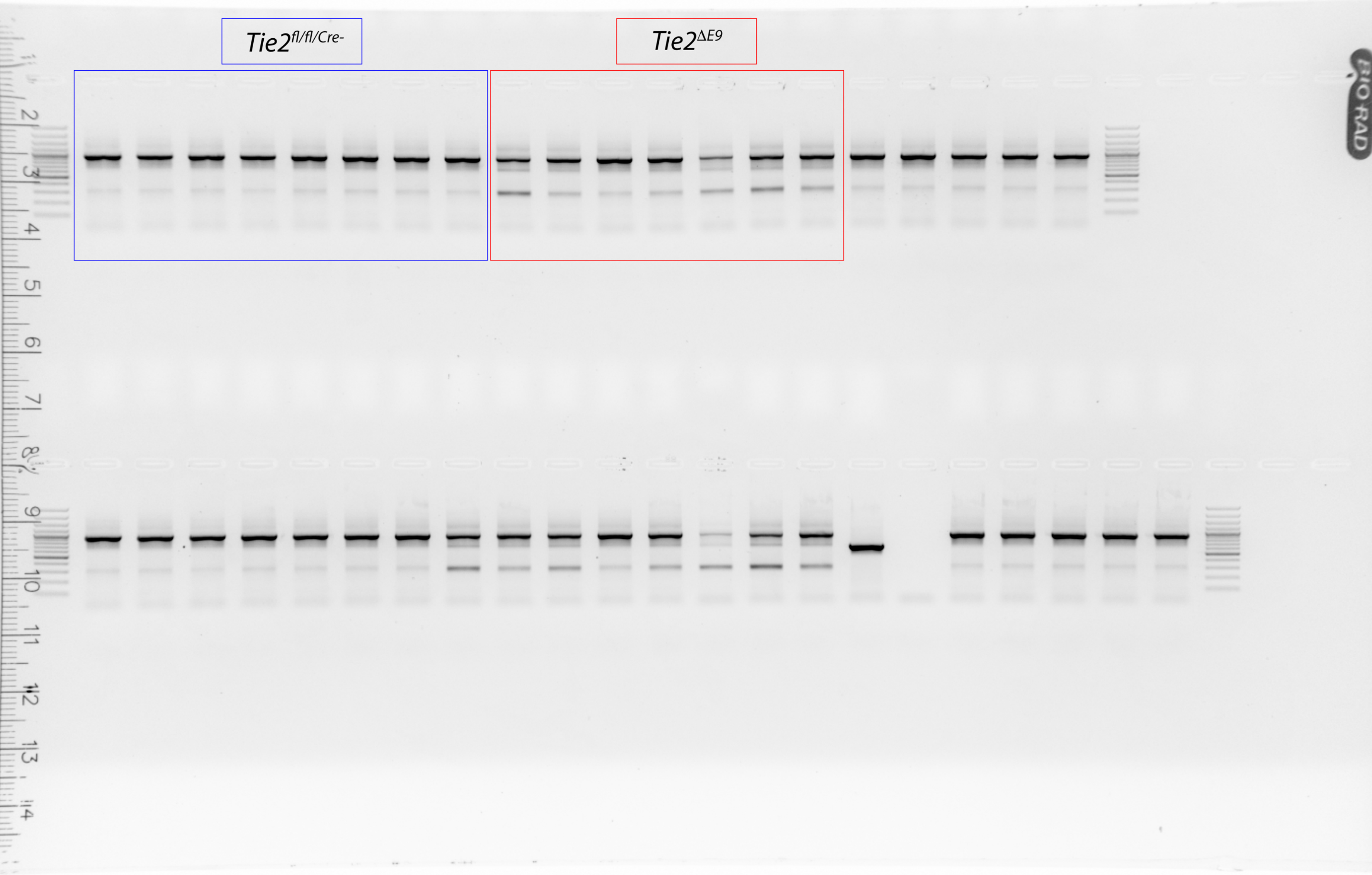

unedited gel S1 Fig - liver
