# Supplementary material for: Pattern of tamoxifen-induced Tie2 deletion in endothelial cells in mature blood vessels using endo SCL-Cre-ERT transgenic mice
Source: PLoS One. 2022 Jun 8;17(6):e0268986. doi: 10.1371/journal.pone.0268986 (PMC9176780; doi:10.1371/journal.pone.0268986)
Supplement: Attachment — Submitted filename: PONE-D-21-04716_Response to Reviewers.docx [file pone.0268986.s018.docx]

Rebuttal for PONE-D-21-04716: “Pattern of inducible endothelial Tie2 knockout in mature mouse microvasculature is organ- and vascular compartment-dependent” **by Zwiers et al.**

We would like to thank the reviewers for their detailed and insightful comments, which we will address below in a point-to-point manner. We also would like to thank the editor for giving us the opportunity to respond to these comments. Due to ongoing COVID-19-related lab restrictions, temporary lack of plastics, and delays in delivery of materials due to Brexit, we needed extended time for the execution of some of the new analyses, the subsequent writing of the rebuttal, and revising the manuscript. We sincerely apologize for this delay.

Tables and Figures included in this rebuttal are marked R and are for rebuttal purposes only, unless otherwise stated. Lines to which we refer can be found in the cleaned-up version of the revised manuscript.

**Reviewer #1.**

**Comment Rev1-1:** Why was brain Tie2 expression upon knockout not examined?

**Response to Rev1-1:** The larger interest of our research revolves around endothelial heterogeneity in organs that are affected by systemic inflammatory processes as prevail in sepsis, endotoxemia, and shock induced by hemorrhage. In previous research, we and other showed that in animal models of these conditions, organ dysfunction and Tie2 loss occurs mainly in lung, kidney, and liver, while the heart and aorta are considered resistant to inflammation and vascular permeability. Indeed, brain can also be considered an organ of interest, yet the vessels in the brain express lower levels of Tie2 compared to the organs reported here. We furthermore lack expertise to analyze the individual components of this large and anatomically complex organ. This led us to exclude this organ from the current study.

**Comment Rev1-2:** Protein levels of Tie1 have to be determined as well. Especially considering quite small degree of mRNA reduction. What is justification behind of selective description of kidney Tie1 mRNA compartmentalization (Figure 5)? Why are other organs not analyzed?

**Response to Rev1-2:** In our hands, small changes in mRNA levels of genes of interest was never associated with quantitatively picking up changes in protein levels when using Western Blot. In addition, we were not able to find commercial ELISA kits for Tie1 quantitation that were validated for analysis of tissue homogenates. Similarly, while considering IHC/IF worthwhile pursuing, as these techniques may also enable identification of possible heterogeneous expression loss of Tie1, we have not been able to find suitable antibodies for this purpose.

As we agree with the reviewer that quantitative localization of Tie1 and the other members of the Ang/Tie system will improve our understanding of local changes in expression in response to (partial) loss of Tie2, we have adapted the Discussion section as follows (Line 404):

Methods that enable the localization and quantitation of the four proteins of the Ang/Tie family studied here, and that can be combined with quantitation of Tie2 phosphorylation in discrete microvascular segments, will be crucial for elucidating how these molecular systems contribute to heterogeneous microvascular endothelial behavior.

Concerning the “selective description of kidney Tie1 mRNA compartmentalization (Figure 5)”: We assume that the reviewer is asking why only arterioles and glomeruli were analyzed. Owing to the physical limitations posed by the size of the laser beam used for Laser Microdissection (LMD), we are able to dissect sufficient numbers of arterioles and glomeruli for subsequent RT-qPCR analyses. However, LMD of microvascular beds from heart and liver sections has so far not provided sufficient material for further analysis. Finally, LMD of microvascular beds from lung sections is virtually impossible, since lung tissue harvested in this study collapsed during excision and snap-freezing.

From our perspective, introducing the (limited) information on two kidney microvascular segments is still valuable for readers, as Tie2 is heavily investigated as target for therapeutic intervention in acute kidney injury (AKI).

**Question Rev1-3:** Discussion section does not provide proper analysis of the reported findings.

**Response to Rev1-3:** We apologize for the fact that we do not fully understand the core criticism of this comment. If the reviewer refers to the fact that we did not discuss the data per figure: our approach is, and has been over the years, to highlight the most important findings in relation to findings published by others, and to discuss methodological issues and cell biological observations that we considered to be of interest for the readers.

In this rebuttal we address some issues that have now been incorporated in the Discussion section of our revised manuscript. Possibly, some of these represent what reviewer 1 identified as lacking.

**Question Rev1-4:** Given the small sample size, were the samples normally distributed? What normality test was used? Result section is incomplete (no information about “means ± SEM or SD” whatsoever).
Figures (plots) do not have any statistical info embedded (no SEM or SD bars).

**Response to Rev1-4:**

We thank the reviewer for this remark, as indeed, the normality test was not mentioned in the original manuscript. We performed a Shapiro-Wilk normality test on mRNA expression data in whole organ extracts and in LMD isolates from the kidney (see Table R1). We inserted a clarifying sentence at line 148-150 in the original manuscript:

Normality of distribution was tested using the Shapiro-Wilk normality test on Tie2 mRNA data obtained from whole organ extracts and LMD isolates from the kidney. The results showed that values are normally distributed.

**Table R1**: *Shapiro-Wilk normality test for Tie2 mRNA level sample distribution in whole organ isolates and glomeruli and arterioles laser microdissected from kidney.*

As suggested by the reviewer, we added SD bars and corresponding information to figures 2, 3B, 4, 5, S2, S3, and S5. As we provide information on means and p-values in the legends, and now also SD values in the figures, we consider mentioning this information in the Results section as redundant.

**Minor questions Reviewer 1:**

**Rev1-5:** Introduction: First paragraph of introduction (lines 42 – 56) would benefit from editing to correct logical and grammar flaws.

**Response to Rev1-5:** Based on this comment we changed the first paragraph as follows (line 43-54):

Tyrosine-protein kinase receptor Tie2, also known as Tunica interna Endothelial cell Kinase, or TEK, is expressed by vascular endothelial cells, as well as other cell subsets of the hematopoietic lineage [1,2]. Endothelial Tie2 plays a prominent role in blood vessel development, vascular integrity, and endothelial responses to inflammation [3–6]. The current model of endothelial Tie2 engagement in signal transduction describes binding of non-endothelially produced agonist angiopoietin (Ang)-1 to Tie2 under quiescent conditions(7, 8). Binding of Ang-1 induces autophosphorylation of Tie2, resulting in PI3-Akt signal transduction and blood vessel stabilization, ensuring vascular integrity [9-–13]. In acute inflammation, such as prevails in, for example, sepsis, endothelially stored Ang-2 is rapidly released from Weibel-Palade bodies and competes with Ang-1 for binding to Tie2 [14–17]. As a result, Tie2 activation is aborted, and endothelial barrier function and vascular integrity are lost. Tie2 signaling is not only controlled by a balance between levels of Ang-1 and Ang-2, but also by heterodimerization of Tie2 with Tyrosine-protein kinase receptor Tie1, which stabilizes Tie2 auto-phosphorylation and downstream signaling [7].

**Rev1-6:** Results/Figures: Panels in Figure 3 need to have scale bars and magnification indicators.

**Response to Rev1-6:** Due to the large number and small size of the photomicrographs in Figure 3A, we were not able to add scale bars to these figures in a legible manner. Instead, we added information about the magnifications used to make the photomicrographs in the legend of Figure 3A (line 246).

**Reviewer #2**

For clarity, we divided the major criticism into two separate parts. The first part deals with the issue whether truncated *Tie2* mRNA is produced when exon 9 is deleted (creating the ΔE9 transgenic). The second part addresses the status of Tie2 protein after exon 9 deletion.

**Question Rev2-1:** Validity of the model for Tie2 deletion – The apparent lack of complete Tie2 deletion in ECs raises doubt about the mouse model. The deletion of exon 9 potentially averts the possibility of interfering with promoter/enhancer elements near the 5’ end of the gene. However, it is possible that this targeting strategy does not completely delete Tie2. If not, it may be detected by the Taqman PCR primer pair used to analyze Tie2 expression, which targets the exon 7-8 boundary. […] To address these issues, it will be important to test another primer pair downstream of the deletion.

**Response to Rev2-1:** We thank the reviewer for this comment. In all honesty, we never considered that a truncated Tie2 product could be formed upon excision of exon 9, as in silico assessment of the consequences of deletion of exon 9 of Tie2 showed a high probability of abrogation of *Tie2* mRNA synthesis and abrogation of protein formation. Moreover, in our previous publication (*Jongman et al, Shock (51); 6; p757-769*) we reported the construction of *Tie2^+/-^* heterozygous mice, for which we crossed the same exon 9 *Tie2*^floxed/floxed^ mice with *Hprt-Cre* mice. This transgenic model was characterized by a 50% reduction in Tie2 mRNA and protein in various organs. Furthermore, F1xF1 litter only consisted of wildtype and *Tie2^+/-^* mice, while homozygous *Tie2^-/-^* mice were not viable. These findings indicate loss of Tie2 upon exon 9 deletion.

Considering the importance of this reviewer’s remarks, we pursued the advice and performed RT-qPCR analyses with two additional Taqman primer/probe sets recognizing *Tie2* mRNA at exon boundary 8/9, respectively 15/16. If upon exon 9 deletion truncated *Tie2* mRNA were formed, one would expect similar levels of mRNA in *Tie2^fl/fl/Cre-^* control and *Tie2^∆E9^* samples using primer/probe set 7/8. Primer/probe sets 8/9 and 15/16 would only yield product in *Tie2^fl/fl/Cre-^* control samples, in which full length *Tie2* mRNA is present.

Figure R1-A shows that the extent of *Tie2* mRNA product formation by the qPCR reaction differed between the 3 primer/probe sets. When applying them to *Tie2^∆E9^* transgenic mouse lungs, all primer/probe sets showed approximately 50% reduction in *Tie2* mRNA compared to control mice (figure R1-B). From these data, which we have now introduced in the revised version of the manuscript as S5 Fig, we conclude that exon 9 deletion does not yield truncated *Tie2* mRNA. *Tie2* mRNA detected in *Tie2^∆E9^* mice with the exon 7/8 primer/probe set used throughout this study, therefore only represents full length *Tie2* mRNA. This can be considered residual and points to heterogeneous knock out of Tie2 in different locations of the microvasculature.

***Figure R1. Tie2 mRNA expression in lungs of control and Tie2 knockout mice determined by use of 3 different primer/probe sets.*** *Tie2 mRNA expression levels were determined in lungs of Tie2^fl/fl/Cre-^ control mice and Tie2^ΔE9^ knockout mice after tamoxifen treatment by RT-qPCR and use of 3 different primer/probe sets that bind at the boundary of Tie2 exon 7/8, exon 8/9, or exon 15/16. (****A)*** *Tie2 expression in Tie2^fl/fl/Cre-^ control, and Tie2^ΔE9^ knockout samples. Exon binding sites of the primer/probe sets are indicated under the X-axis.* *The graph shows individual values and means ± SD.* *Closed circles: Tie2^fl/fl/Cre-^ mice (n=8); open circles: Tie2^ΔE9^ mice(n=7)* ***(B)*** *Percent knockout of Tie2 mRNA* *in Tie2^ΔE9^ knockout samples relative to control mice. Bars shows means ± SD.*

The following revisions have been made in the manuscript:

In the Materials and Methods section we inserted relevant exon 8/9 and exon 15/16 primer/probe set information in Table 1 (RT-qPCR primers), line 113:

| *Tek* | Mm00443243_m1 | Tyrosine-protein kinase receptor (Tie2), CD202 (exon 8/9) |
| --- | --- | --- |
| *Tek* | Mm01256894_m1 | Tyrosine-protein kinase receptor (Tie2), CD202 (exon 15/16) |

We also added the following sentence (line 103):

To determine the possible formation of truncated Tie2 mRNA upon exon 9 deletion, we used 3 different primer/probe sets that hybridize at cDNA sites before (at the boundary between exon 7 and exon 8, hereafter referred to as ‘exon 7/8’), at (exon 8/9), or after exon 9 (exon 15/16) (see Table 1).

**Question Rev2-2:** Furthermore, there may be residual protein expression, which may be detected by an antibody directed against the extracellular domain of Tie2, which TEK4 and the R&D ELISA appear to do. Without addressing this issue, it is difficult to draw any conclusions about the authors observations regarding effects on expression of Tie2 and the other Tie family genes/proteins.

**Response to Rev2-2:** The Tie2 antibody used for IHC staining indeed detects the extracellular domain. Although R&D systems does not reveal the exact identity of the capture antibody in its ELISA kit (Mouse Tie-2 Immunoassay, MTE200 of R&D Systems), the fact that the ELISA is widely used to quantitate soluble Tie2, makes it safe to conclude that it also detects the extracellular domain of Tie2.

Although highly unlikely, as discussed in Response to Rev2-1, if exon 9 deletion resulted in the formation of a truncated Tie2 protein, this protein would only comprise the extracellular domain, and thus would be much smaller than the full length protein. To investigate whether such smaller Tie2 proteins had formed in the transgenic mice, we subjected lung homogenates *of Tie2^fl/fl/Cre^ and Tie2^∆E9^* mice to Western Blot. Proteins were detected using two antibodies: Ab33 from Upstate detects the extracellular domain of Tie2, and an Ab from Cohesion Bioscience recognizes Tie2 p-tyrosine at position 1108, i.e., the intracellular domain. Although smaller bands are visible at ~100kD, 45kD, and 20kD, these bands are not restricted to the samples of the *Tie2^∆E9^* mice (Figure R2), indicating that they do not represent truncated Tie2 protein formed as a result of deletion of exon 9.

***Figure R2: Determination of the molecular weight of Tie2 protein in mouse lungs by Western Blot.*** *Briefly, lung protein isolates of Tie2^fl/fl/Cre-^control and Tie2^∆9^ knockout mice (n=3 per group, 25ug per sample), were run on a 10% SDS gel, proteins were transferred to a nitrocellulose membrane, and detected with Tie2 Ab33 (cat no 05-584, Upstate®, Merck (Sigma Aldrich), Amsterdam, The Netherlands) and* *Tek C-Term, pY1108 (cat no. ABIN2705294, Cohesion Biosciences, London, UK).*

One could argue that truncated Tie2 protein, since it has lost its transmembrane domain, would not be detectable in organs but rather in a soluble form in plasma. To assess whether this is the case upon exon 9 deletion, we analyzed plasma from *Tie2^+/-^* heterozygous mice (referred to above) that were bred by crossing *Tie2^floxed/floxed^* exon 9 mice with *Hprt-Cre* mice. In these *Tie2^+/-^* mice, sTie2 levels in plasma did not increase, but instead were 50% lower than those in wildtype littermates (Figure R3), supporting the conclusion that excision of exon 9 of Tie2 does not result in truncated Tie2 protein.

***Figure R3:*** ***Plasma levels of soluble Tie2 in heterozygous Tie2^+/-^ mice and wild type littermates****. Tie2 was analyzed by ELISA in plasma of heterozygous Tie2^+/-^ mice generated by crossing exon 9 Tie2^floxed/floxed^ mice with Hprt-Cre mice. Plasma of the resulting F1 litter was analyzed (n=6, mean values +/- SD). (unpublished representation of plasma data reported by Jongman et al, Shock (51); 6; p757-769).*

Based on the above, we conclude that Tie2 mRNA and protein detected in the *Tie2^∆E9^* mice reported in our manuscript, represent full length, residual Tie2, rather than truncated mRNA/protein. We have adapted the revised manuscript as follows:

In the Results section (line 204-213):

To confirm that reduced levels of *Tie2* originate from residual of full length *Tie2* and is not a result of the formation of truncated *Tie2*, we performed RT-qPCR analysis on lung RNA isolates from *Tie2^fl/fl/Cre-^* control mice and *Tie2^∆E9^* knockout mice. For this analysis we used three primer/probe sets that bind to different intron-overspanning regions within the *Tie2* cDNA. While they hybridized to the Tie2 cDNA to different extents, the percentage of *Tie2* downregulation in the *Tie2^∆E9^* knockout mice versus controls measured by each of these primer/probe sets was approximately 50% and comparable to one another (S5 Fig). We also performed Western Blot analysis on protein isolates from the lung, and did not detect small size, truncated Tie2 protein in *Tie2^∆E9^* knockout mice compared to controls (data not shown). Based on these results we concluded that the decrease in Tie2 mRNA and protein levels in our mouse model represents residual full length Tie2*.*

In the Discussion, we changed and added the following (line 348):

An important observation in our *Tie2^ΔE9^* knockout model is that reduction of Tie2 did not exceed 70-85% of control values in whole organs or in specific microvascular compartments. The residual 15-30% Tie2 mRNA and protein is full length Tie2, produced by cells in which deletion of exon 9 did not take place.

**Question Rev2-3:** The discrepant finding that Tie2 mRNA is persistently expressed in arterioles (Fig. 5B) whereas protein expression is not (Fig. 3A) leads the authors to invoke miRNAs, when in fact the explanation may be much simpler, as noted above. Moreover, it is not clear why miRNAs would be upregulated preferentially in the knockout vs. the wild-type mouse…[….]….it would also be important to demonstrate absence of protein expression by western blotting of tissues (or isolated cells) to determine whether a truncated protein product is expressed as a result of the deletion.

**Response to Rev2-3:** At present, it is technically impossible to quantitate Tie2 protein by Western Blot or ELISA in cells isolated from specific microvascular segments by enzymatic digestion or laser microdissection, due to lack of markers to identify these subsets, respectively limited yield of material in the LMD procedure. The additional data generated and added to the revised manuscript showing that Tie2 mRNA and protein in the Tie2^∆E9^ knockout mice is full length residual Tie2, made us conclude that right now the main outcome of our study firmly stands, namely that in our new model of TMX-induced endothelial knockout of Tie2, some vessels show a high extent of protein loss, while others are hardly affected - whatever the molecular reason is.

We agree with the reviewer that our explanation of why in some cases protein levels of Tie2 in dedicated microvascular segments are reduced, while mRNA levels are unchanged, is speculative. We have revised this paragraph as follows, to make this clear (line 368-378):

Zooming in on the microvasculature of the kidney we found a discrepancy between unaltered *Tie2* mRNA expression, and strongly reduced Tie2 protein expression in the arteriolar compartment of knockout mice. At present, this finding cannot be molecularly explained. Possibly, smooth muscle cells that support renal arterioles express *Tie2* mRNA (comparable to *Tie2* expression by pericytes in mice) [Teichert et al, Nat. Commun. 2017;8: 16106], and Tie2 expression in smooth muscle cells in rat aorta [Iurlaro et al, J. of Cell Science 2003;116: 3635-3543], yet do not translate this into protein (Fig 3A) due to the presence of microRNAs that can silence Tie2 protein formation [Besnier et al, Mol. Ther. - Nucleic Acids, 2019;17: 49–62]. Since laser microdissection enriches samples for endothelial cells but never yields a completely pure endothelial sample, *Tie2* mRNA produced by smooth muscle cells may have contributed to the *Tie2* mRNA signal in these samples. Such an explanation, however, remains hypothetical as Tie2 expression by smooth muscle cell in arterioles in mouse kidney has not been reported. Mechanisms of Tie2 protein expression regulation within specific vascular compartments within an organ require further investigation.

**Question Rev2-4:** Relative inefficiency of the SCL-Cre-ERT driver – The apparent “heterogeneity” of Tie2 deletion may just be due to poor excision from this Cre driver, which has been shown to be as low as 60% efficient. The fact that Hprt-Cre-mediated deletion of Tie2 in the authors’ earlier paper resulted in embryonic lethality suggests that the targeting approach works. Thus, poor deletion with this Cre driver might result in a poor model for any pathophysiological studies. Although such pathological models may be beyond the scope of this paper, it is easy to conclude that this model might have limited efficacy without demonstration otherwise. The use of a reporter line, such as an SCL-Cre-ERT;ROSA-TdTomato line, to demonstrate simultaneously where the Cre is being expressed along with residual Tie2 co-expression would be helpful. This is particularly relevant in the kidney, where there is apparently such high residual Tie2 expression.

**Response to Rev2-4:** The choice of the SCL-Cre-ERT driver was based on the publication by Göthert et al. (Blood 2004;104; p1768-1777), who showed high penetrance of Cre expression in the microvasculature of several organs using an SCL-Cre-ERT LacZ reporter mouse. Our investigation aimed to apply this technology to knock out endogenous Tie2. The results presented in this paper lead us to conclude that a direct relation between Cre-based LacZ expression and Cre-based Tie2 knockout cannot be established. Using an additional reporter line, as proposed by the reviewer, is interesting from the point of view that it would provide experimental data explaining the heterogeneous loss of Tie2, yet will not change the outcome of the model. We would like to stress the fact that by now it is broadly accepted that none of the endothelial-specific driver models accomplish 100% knockout in all blood vessels in all organs, as summarized by Payne et al (ATVB 2018; 38; p2550-2561).

We do not agree with this reviewer’s conclusion that this model might have limited efficacy, since critical analyses of the (micro)vascular beds within the organs studied in this manuscript revealed an 80% reduction in Tie2 expression in several of these. Still, each organ and microvascular bed should be investigated for extent of knockout before any (mechanistic and/or pathophysiology related) conclusions can be drawn. This recommendation not only holds true for SCL-Cre-ERT driver models, but for any model, as we discuss in line 412-416 of the revised manuscript.

**Question Rev2-5:** Effects of Tie2 deletion on expression of other genes – The data suggest that expression of Tie1, Ang1, and Ang2 is variably altered by Tie2 deletion. However, the veracity of these data is not clear given the questions about residual Tie2 expression, and this impacts the correlations in Fig 5C. Moreover, many of the statistically significant changes in gene expression appear trivial, such as the 12% reduction in Tie1 mRNA and the 17% increase in Ang2 expression in Tie2-deficient hearts (Fig 4A,C), thus it is not clear whether these changes have any biological significance. Without testing this in some physiological or pathophysiological model, as suggested in point #2, the relevance of these findings remains unclear.

**Response to Rev2-5:** In the manuscript we showed mRNA levels based on assessment with a primer/probe pair for the E7/E8 boundary. In our ‘Response to Rev2-1’ we demonstrated that these levels reflect full length residual *Tie2*. Therefore, the observations in the correlation plots in Fig 5C are considered relevant.

We agree that the marginal increase in renal arteriolar *Tie1* and *Ang2* require further investigation to reveal whether this finding is biologically relevant, and calls for further studies. We have added the following to the Discussion section to highlight this ( line 402-407):

Similarly enigmatic is the observation that in glomeruli of transgenic mice, the balance in Tie2 /Tie1 expression, such as is present under control conditions, is altered. No such alterations were present in arterioles. Methods that enable the localization and quantitation of the four proteins of the Ang/Tie family studied here, and that can be combined with quantitation of Tie2 phosphorylation in discrete microvascular segments will be crucial for elucidating how these molecular systems contribute to heterogeneous microvascular endothelial behavior.

**Minor comment Reviewer 2:**

**Rev2-6:** Tie2 nomenclature (line 42) – The original name for Tie2 was Tunica interna Endothelial cell Kinase, or TEK (still the primary gene name) (Dumont, Oncogene, 1992). If the authors wish to acknowledge any name besides TIE2, it is suggested that this names be used, as “Tyrosine kinase receptor 2” is generic and not an accepted alias, although the related “Tyrosine-Protein Kinase Receptor TIE-2” is listed in GeneCards.

**Response to Rev2-6:** “Tyrosine kinase receptor 2” of the original manuscript was replaced by “Tyrosine-protein kinase receptor Tie2, also known as Tunica interna Endothelial cell Kinase or TEK” in the revised version of the manuscript at line 27

**Reviewer #3:**

**Suggestion Rev3-1:** From what I gather from the Methods, the animals were injected with TMX 3 times, once each in 3 successive weeks, at the dose listed, but at what age did the injections start? Whether this is an adult-age deletion or early-postnatal is critical to all of the results, but this information was not given. But this information has to be clearly stated in Methods. And equally importantly, since, the conclusions drawn depend on the age of deletion, the Discussion include comments on what this means in light of whether this was adult or early postnatal deletion (or other).

**Response to Rev3-1:** The interest of our research focuses on microvascular endothelial heterogeneity in adult tissues, on how intrinsic heterogeneity relates to differences in responses of microvascular segments to disease conditions, and on what the pharmacological consequences thereof are.

For this reason, we worked with adult mice that were between 10-24 weeks of age when they received the first tamoxifen i.p. injection. We have stated this information in the Materials and Methods section, line 82/83 and 91/92.
