# Supplementary material for: Pattern of tamoxifen-induced Tie2 deletion in endothelial cells in mature blood vessels using endo SCL-Cre-ERT transgenic mice
Source: PLoS One. 2022 Jun 8;17(6):e0268986. doi: 10.1371/journal.pone.0268986 (PMC9176780; doi:10.1371/journal.pone.0268986)
Supplement: Attachment — Submitted filename: PONE-D-21-04716_Response to Reviewers.docx [file pone.0268986.s019.docx]

Rebuttal for PONE-D-21-04716: “Pattern of inducible endothelial Tie2 knockout in mature mouse microvasculature is organ- and vascular compartment-dependent” **by Zwiers et al.**

We would like to thank the reviewers for their time invested to enable us to further improve our manuscript, and the editor for giving us the opportunity to respond to the comments and questions raised.

**Reviewer #1.**

**Comment Rev1-1:** Authors did not properly address critical comments provided for previous version. Manuscript is still lacking methodological rigor, results are presented in poor manner and conclusions expressed in the discussion are not supported by findings.

**Response to Rev1-1:** In the first round of review, we extensively addressed the issues brought up by reviewer #1. Summarizing, we described choices regarding experimental design, including the lack of availability of antibodies to stain Tie1 protein in mouse tissues, amended the manuscript with requested additional statistical information, and improved textual parts of the manuscript. As the reviewer does not detail which issues were not properly addressed, it is not possible for us to (re)act. Furthermore, the criticism brought up by this reviewer is not shared by the other two reviewers. Instead, they assess the manuscript as technically sound, the data supporting conclusions (except for issues raised by reviewer #2 which we address below), the statistical analyses appropriate and rigorous, and the manuscript presented in an intelligible fashion.

**Reviewer #2.**

**Comments Rev2-1:** I agree from the responses to my prior concerns about truncated RNA and protein that the model works, i.e., that Tie2 is targeted by the exon 9 deletion, but it is clear that the deletion is inefficient and that this is due to inefficient Cre-mediated excision of Tie2 exon 9 with the SCL-Cre driver.

**Response to Rev2-1:** We are happy to read that, based on the additional data generated and added to the revised version of the manuscript, the reviewer agrees that the conclusion is justified that the inducible, endothelial cell specific Tie2 knockout model works.

**Comment Rev2-2**:   However, there still seems to be an argument by the authors that this could be a good model for pathophysiological studies. That may be true, but when the western blot in Fig R2 shows almost no loss of Tie2 expression in lung (albeit with no loading control shown), I have to question this conclusion, irrespective of higher apparent levels of deletion in microvascular beds. If I saw that western as evidence of the validity of the model, I would conclude that there was no deletion.

**Response to Rev2-2**: We would like to stress that Figure R2 in our initial rebuttal aimed to answer this reviewer’s question whether truncated protein had been formed upon TMX treatment. Accordingly, the Western Blot analysis of Tie2 protein was designed to optimally detect intact Tie2 protein and potential truncation fragments thereof. From this data we concluded that no truncated protein was produced, which, in combination with PCR analyses using different primer sets, made us, and this reviewer, conclude that the mouse model works.

Figure 2 in the manuscript presents the quantitation of Tie2 protein by ELISA, which is the broadly accepted, superior technique for this purpose. The data in Figure 2 revealed loss of Tie2 protein in lungs, heart and aorta of TMX-treated animals. These quantitative data next led us to perform immunohistochemical staining of Tie2 in the organs, to identify which endothelial cells had lost the protein. We were as surprised as this reviewer to see the heterogeneous patterning of Tie2 loss shown in Fig 3A, and were even more surprised that this heterogeneous loss was highly consistent, as shown in figure 3B.

In our search for published studies reporting TMX-inducible endothelial knockout mouse models to compare our data with, we discovered that in many papers, knockout was proven by exposing endothelial cells isolated from WT and TG animals, to OH-TMX *in vitro,* without providing further knockout evidence *in vivo*, or by analysis of target gene knockout in endothelial cells in one organ, while (patho)physiology studies focussed on another organ or other vascular segment [1–4]. Other studies used a reporter model to demonstrate the vascular beds in which the driver for Cre was activated by TMX, yet failed to report whether the target protein of interest was indeed knocked out in endothelial cells in these vascular beds [1,5].

As we consider quantitation of target gene knockout when using such models of importance for the reader, we briefly discuss our outcomes in the context of what others have published in the Discussion section:

**Line 369:** Combining *Tie2^floxed/floxed^* transgenic mice with *end-SCL-Cre-ER^T^* transgenic mice resulted in variable, yet highly reproducible Tie2 knockout, which was dependent on the location of the endothelial cells in the microvasculature in organs. Future studies will assess whether crossing of the *Tie2^floxed/floxed^* mouse with e.g., *Cdh5Cre^ERT2^* or *Flk-1-Cre* mice [37, 38] yields knockout of Tie2 to the same extent, and in a similar pattern, as we report here. Knocking out another gene in the same Cre-driver mouse may result in different patterns of target gene loss [35,36,39]. Important to note is that many studies rely on reporter models to demonstrate where Cre recombinase is active, show target gene knockout in an organ which is not the organ under investigation for functional studies, or only show target gene knockout by *in vitro* exposure to tamoxifen. Information about the extent and location of target gene knockout is the cornerstone of this kind of studies to safeguard validity of the conclusions drawn, especially since extensive molecular heterogeneity exists between endothelial cells [40,41] which can have major functional consequences [42].

An elegant paper by Koh et al [6] shows in a Cdh5-Cre-ERT mouse model that 85% knockout of Tie2 in the brains of mice has occurred after short term treatment with TMX when these mice are 8 weeks old. They next study whether Tie2 has a role in a particular pathophysiological process in the brain. This study is an exception to the examples referred to above, as it demonstrates loss of target protein in the organ under study in a pathophysiological process. The mouse model Koh et al. use is the model reported by Savant et al., in which in the lungs of mice 6 days after birth, 25-33% Tie2 knockout had occurred using a similar TMX dosing regimen [4]. We added this information to the existing paragraph, discussing the previously reported study by Savant on endothelial knockout of Tie2 using Cre recombinase:

**Line 398**: An elegant study employing the same mouse model to investigate whether Tie2 exerts a role in tissue repair after photothrombotic injury evoked in the brain of 8 week old mice, showed 85% reduction of Tie2 protein expression in the brain [45]. It may well be that also in this *Cdh5Cre^ERT2^* mouse model the extent of knockout of Tie2 depends on the organ.

**Comment Rev2-3**:  I’m having difficulty determining the main point of this paper.

**Response to Rev2-3:** The main point of this paper, is that knocking out Tie2 in endothelial cells *in vivo* using the SCL-Cre-ERT system leads to variation in extent of knockout in organs and microvascular segments in these organs. A corollary to this is that meticulous analysis of where the loss of target protein happens is crucial for this model, to prevent wrongful extrapolation from one organ to the other, or from one vessel to the other, a notion supported by this reviewer. We agree to the fullest with this reviewer that this ‘seems obvious, as such an approach is a critical part of demonstrating the validity of any model’, yet, as described in **‘Response to Rev2-2’**, many studies, also using other drivers than SCL, steer away from demonstrating target gene knockout in the microvascular bed studied in (patho)physiological processes. We expect that the main point of our manuscript will create awareness regarding the importance of analysing the extent and location of knockout, and have added a paragraph on this matter to the Discussion section (**line 392-401**).

**Comment Rev2-4**:  The authors state that the SCL-Cre driver was chosen because of its apparently high efficiency based on the prior study using a LacZ reporter: “The results presented in this paper lead us to conclude that a direct relation between Cre-based LacZ expression and Cre-based Tie2 knockout cannot be established.” That may well be true, but the best approach to establish the model’s efficiency would be to use simultaneous Cre-mediated reporter expression, as suggested in the first review. I recognize that this is likely beyond the scope of this report […].

**Response to Rev2-4**:  Investigating mechanisms to explain the heterogeneous patterning of Tie2 loss was, and is, indeed, beyond the scope of our study.

Yet, based on this reviewer’s comment, we tried to get a glimpse of the location of activated, i.e., nuclear Cre recombinase, and to relate this to Tie2 expression. For this purpose, we fluorescently double-stained kidney sections of C57BL/6 wildtype mice, TMX-treated *Tie2^fl/fl/Cre-^* control mice, and TMX treated *Tie2^ΔE9^* knockout mice, with anti-Cre and anti-Tie2 antibodies. We used two different anti-Cre-recombinase antibodies, i.c., Rabbit polyclonal Ab40011 (Abcam, Cambridge, UK) and Rabbit polyclonal cat#908001 (BioLegend, San Diego, USA). Unfortunately, both antibodies showed significant non-specific binding in all three groups of mice, which we were not able to block (data not shown). We could therefore not draw any conclusions on the exact location of Cre-recombinase and Tie2 deletion in *Tie2^ΔE9^* knockout mice, to explain, at least in part, the observed heterogeneous pattern of Tie2 knockout.

**Comment Rev2-5**:  In the revised discussion, the authors have done a good job of discussing the limitations of various Cre drivers. […] To me, the simple conclusion from this paper is that this particular targeted mutation paired with this Cre driver is variably efficient in deleting Tie2. If claims beyond this regarding the efficacy of the model are to be made, it would be important to show simultaneous co-expression of a reporter, as suggested, or perhaps just use a different Cre driver (such as VE-cadherin-Cre).

**Response to Rev2-5**: We agree with this reviewer that we cannot and should not extrapolate our findings to any other mouse model, and therefore have carefully rephrased generalized statements into dedicated information (highlighted yellow in the revised manuscript) about the model which we here report:

**Line 319**: For this purpose, we used the *end-SCL-Cre-ER^T^* mouse model, in which tamoxifen-inducible Cre ER^T^ recombinase expression was driven by the endothelial enhancer of the Stem Cell Leukemia locus. In this mouse model, using LacZ as reporter, Cre recombinase was shown to be active in the microvasculature of several, though not all, organs [24].

We furthermore heavily revised the section on using other driver-Cre mouse models, as described in our ‘Response to Rev2-2’ (**line 369-380 and 392-401**).

**Comment Rev2-6**:  Accordingly, the title is misleading, as it suggests that the heterogeneity is somehow Tie2-dependent rather than being Cre-dependent. A better title would be simply, “Pattern of endothelial Tie2 deletion in an SCL-Cre-ERT;Tie2floxed mouse line”.

**Response to Rev2-6:** We have adapted the title as advised by the reviewer: Pattern of tamoxifen-induced Tie2 deletion in endothelial cells in mature blood vessels using endo SCL-Cre-ER^T^ transgenic mice.

**Reviewer #3.**

**Reviewer #3** judges the manuscript technically sound, with data supporting the conclusions, with statistical analyses performed appropriately and rigorously, and presented in an intelligible fashion and written in standard English and has no further comments.

**References**

1.	Korhonen H, Fisslthaler B, Moers A, Wirth A, Habermehl D, Wieland T, et al. Anaphylactic shock depends on endothelial Gq/G11. J Exp Med. 2009;206: 411–420. doi:10.1084/jem.20082150

2.	Langer HF, Orlova V V., Xie C, Kaul S, Schneider D, Lonsdorf AS, et al. A Novel Function of Junctional Adhesion Molecule-C in Mediating Melanoma Cell Metastasis. Cancer Res. 2011;71: 4096–4105. doi:10.1158/0008-5472.CAN-10-2794

3.	Schmidt TT, Tauseef M, Yue L, Bonini MG, Gothert J, Shen T-L, et al. Conditional deletion of FAK in mice endothelium disrupts lung vascular barrier function due to destabilization of RhoA and Rac1 activities. Am J Physiol Cell Mol Physiol. 2013;305: L291–L300. doi:10.1152/ajplung.00094.2013

4.	Savant S, La Porta S, Budnik A, Busch K, Hu J, Tisch N, et al. The Orphan Receptor Tie1 Controls Angiogenesis and Vascular Remodeling by Differentially Regulating Tie2 in Tip and Stalk Cells. Cell Rep. 2015;12: 1761–1773. doi:10.1016/j.celrep.2015.08.024

5.	Perry HM, Huang L, Ye H, Liu C, Sung SJ, Lynch KR, et al. Endothelial Sphingosine 1‑Phosphate Receptor‑1 Mediates Protection and Recovery from Acute Kidney Injury. J Am Soc Nephrol. 2016;27: 3383–3393. doi:10.1681/ASN.2015080922

6.	Koh BI, Lee HJ, Kwak PA, Yang MJ, Kim J-H, Kim H-S, et al. VEGFR2 signaling drives meningeal vascular regeneration upon head injury. Nat Commun. 2020;11: 3866. doi:10.1038/s41467-020-17545-2
