# Supplementary material for: Pattern of tamoxifen-induced Tie2 deletion in endothelial cells in mature blood vessels using endo SCL-Cre-ERT transgenic mice
Source: PLoS One. 2022 Jun 8;17(6):e0268986. doi: 10.1371/journal.pone.0268986 (PMC9176780; doi:10.1371/journal.pone.0268986)
Supplement: Attachment — Submitted filename: PONE-D-21-04716_Response to Reviewers.docx [file pone.0268986.s020.docx]

Rebuttal for PONE-D-21-04716: “Pattern of tamoxifen-induced Tie2 deletion in endothelial cells in mature blood vessels using endo SCL-Cre-ERT transgenic mice” **by Zwiers et al.**

We would like to thank the reviewers for their time invested to enable us to further improve our manuscript, and the editor for giving us the opportunity to respond to the comments and questions raised.

**Journal Requirements:**

**1-1:** Please address the comments from reviewer 1 regarding reporting of statistics/data availability.

**Response to journal requirements:** To fulfill the journal requirements and addressing the comments by reviewer 1 regarding reporting of statistics and data availability we have uploaded all GraphPad files as supplementary data as well as the raw Excel data files that belong to the ELISA read-out. We have amended the manuscript text as well as the supplementary data titles accordingly.

**Line 128**: Raw data files are available in supplementary data files (S9 Dataset).

**Line 160**: All GraphPad PZFX-files are available as supplementary data (S10-16 Dataset).

**Line 659-676**: **S9 Dataset**. Excel data file that represents all raw data points of the Tie2 ELISA used for Fig 2B. Standard curve is shown for samples measured in one run.

**S10 Dataset**. GraphPad data file used for the statistical analysis of mRNA expression levels as presented in Fig 2A, Fig 4A-C, and S4 Table.

**S11 Dataset**. GraphPad data file used for the statistical analysis of Tie2 protein levels (ELISA) as presented in Fig 2B.

**S12 Dataset**. GraphPad data file used for the statistical analysis of Tie2 protein localization in different organs (IHC) by morphometrics as presented in Fig 3B.

**S13 Dataset**. GraphPad data file used for the statistical analysis of mRNA expression levels in laser microdissected compartments as presented in Fig 5B-E.

**S14 Dataset**. GraphPad data file used for the statistical analysis of Tie1-Tie2 correlations in laser microdissected compartments as presented in Fig 5F,G.

**S15 Dataset**. GraphPad data file used for the statistical analysis of VE-cadherin and CD31 mRNA expression levels as presented in S3 Fig.

**S16 Dataset**. GraphPad data file used for the statistical analysis of Tie2/Tie1 and Ang1/Ang2 mRNA level ratios as presented in S6 and S7 Tables.

**S17 Dataset**. GraphPad data file used for the statistical analysis of Tie2 mRNA expression levels using 3 different primer/probe sets as presented in S5 Fig.

**1-2:** Please review your reference list to ensure that it is complete and correct. If you have cited papers that have been retracted, please include the rationale for doing so in the manuscript text, or remove these references and replace them with relevant current references. Any changes to the reference list should be mentioned in the rebuttal letter that accompanies your revised manuscript. If you need to cite a retracted article, indicate the article’s retracted status in the References list and also include a citation and full reference for the retraction notice.

**Response to review reference list:** We have checked all the references we have used in this manuscript. The list is complete, none of the cited papers have been retracted, hence we did not make any changes.

**Reviewer #1.**

**Comment Rev1-1:** Results section is still incomplete. Authors state that data is presented as mean and SD, however no actual values are included in Results section or Figure legends.

**Response to Rev1-1:** We indeed did not present the data as mean values explicitly in the paper as we judged that this might compromise the readability of the paper. SD however is shown in each figure by error bars and mentioned as such in figure legends. Since reviewer #1 judges loss of readability not an issue, we have included the mean, SD, and *p* values and we adapted the text in the Results section according to her/his advice. Furthermore we have added the following to all relevant figure legends (changes in highlights): ”Graphs show individual values and means (black lines) ± SD (error bars)”.

**Line 205-214**: In *Tie2^ΔE9^* knockout mice, *Tie2* mRNA expression was significantly reduced in kidney (25%; mean *Tie2^ΔE9^*= 0.59 (SD 0.08); mean *Tie2^fl/fl/Cre-^*= 0.79 (SD 0.12); *p=* 0.0026), lung (55%; mean *Tie2^ΔE9^*= 0.12 (SD 0.05); mean *Tie2^fl/fl/Cre-^*= 0.27 (SD 0.06); *p=* 0.0002), heart (35%; mean *Tie2^ΔE9^*= 0.15 (SD 0.03); mean *Tie2^fl/fl/Cre-^*= 0.23 (SD 0.03); *p<*0.0001), and aorta (38%; mean *Tie2^ΔE9^*= 0.13 (SD 0.07); mean *Tie2^fl/fl/Cre-^*= 0.21 (SD 0.04); *p=* 0.013), but not in liver (mean *Tie2^ΔE9^*= 0.17 (SD 0.04); mean *Tie2^fl/fl/Cre-^*= 0.18 (SD 0.05); *p=* 0.54) (Fig 2A). This organ-specific reduction of *Tie2* mRNA was paralleled by a reduction in Tie2 protein in lung (66%; mean *Tie2^ΔE9^*= 2.45 (SD 1.10); mean *Tie2^fl/fl/Cre-^*= 7.21 (SD 3.01); *p=* 0.0017), heart (37%; mean *Tie2^ΔE9^*= 0.31 (SD 0.05); mean *Tie2^fl/fl/Cre-^*= 0.50 (SD 0.06); *p<*0.0001), and aorta (51%; mean *Tie2^ΔE9^*= 0.36 (SD 0.18); mean *Tie2^fl/fl/Cre-^*= 0.73 (SD 0.22); *p=* 0.0033), as assessed by ELISA on whole organ homogenates (Fig 2B).

**Line 241-254**: When Tie2 protein staining was quantified by morphometry in whole organ sections of *Tie2^ΔE9^* knockout and *Tie2^fl/fl/Cre-^* control mice, a statistically significant Tie2 protein reduction was identified in lung (79%; mean *Tie2^ΔE9^*= 3.50 (SD 2.83); mean *Tie2^fl/fl/Cre-^*= 16.52 (SD 5.96); *p=* 0.0002) and aorta (57%; mean *Tie2^ΔE9^*= 2.34 (SD 0.98); mean *Tie2^fl/fl/Cre-^*= 5.45 (SD 1.83); *p=* 0.0015) (Fig 3B, ‘total’).

We next refined quantification of Tie2 protein by selectively focusing on microvascular compartments (Fig 3B). This approach uncovered a significant reduction in Tie2 protein expression in arterioles (70%; mean *Tie2^ΔE9^*= 8.27 (SD 4.94); mean *Tie2^fl/fl/Cre-^*= 27.81 (SD 7.46); *p<*0.0001) and glomeruli (17%; mean *Tie2^ΔE9^*= 35.84 (SD 5.36); mean *Tie2^fl/fl/Cre-^*= 43.04 (SD 4.75); *p=* 0.0163) of the kidney, which had been concealed by whole-tissue quantification. Moreover, a significant reduction in Tie2 protein was detected in arterioles of the heart (83%; mean *Tie2^ΔE9^*= 1.86 (SD 1.08); mean *Tie2^fl/fl/Cre-^*= 10.91 (SD 8.93); *p=* 0.02), which, similarly, had been obscured by whole-tissue quantification. Vascular compartment-specific quantification in lung revealed a significant reduction of Tie2 protein in both arterioles (70%; mean *Tie2^ΔE9^*= 13.51 (SD 5.41); mean *Tie2^fl/fl/Cre-^*= 45.24 (SD 8.05); *p<*0.0001) and capillaries (85%; mean *Tie2^ΔE9^*= 3.01 (SD 4.22); mean *Tie2^fl/fl/Cre-^*= 20.18 (SD 7.45); *p=* 0.0001) (Fig 3B).

**Line 277-287**: In *Tie2^ΔE9^* knockout mice, *Tie1* mRNA expression was significantly reduced in kidney (19%; mean *Tie2^ΔE9^*= 0.40 (SD 0.07); mean *Tie2^fl/fl/Cre-^*= 0.49 (SD 0.08); *p=* 0.0303), lung (23%; mean *Tie2^ΔE9^*= 0.16 (SD 0.01); mean *Tie2^fl/fl/Cre-^*= 0.21 (SD 0.03); *p=* 0.0012) and heart (12%; mean *Tie2^ΔE9^*= 0.35 (SD 0.04); mean *Tie2^fl/fl/Cre-^*= 0.40 (SD 0.03); *p=* 0.0155) (Fig 4A), *i.e.* in organs that also exhibited a significant reduction in *Tie2* mRNA levels. In contrast, the significant *Tie2* mRNA reduction in aorta of *Tie2^ΔE9^* knockout mice was not accompanied by a reduction in *Tie1* mRNA expression. *Tie1* mRNA expression in liver did not differ between genotypes.

Endothelial *Tie2^ΔE9^*knockout did not affect *Ang1*- or *Ang2* mRNA expression in most organs (Figs 4B and C), with the exception of the heart, where *Ang2* mRNA expression was significantly increased (17%; mean *Tie2^ΔE9^*= 0.0066 (SD 0.0007); mean *Tie2^fl/fl/Cre-^*= 0.0056 (SD 0.0008); *p=* 0.0252) in *Tie2^ΔE9^* knockout mice (Fig 4C).

**Line 310-323**: Deletion of *Tie2* exon 9 was accompanied by significantly reduced *Tie2* mRNA levels in glomeruli (mean *Tie2^ΔE9^*= 0.80 (SD 0.14); mean *Tie2^fl/fl/Cre-^*= 1.01 (SD 0.13); *p=* 0.009). In arterioles from control mice high variation in Tie2 expression was observed, with no statistically significant differences between the two groups (mean *Tie2^ΔE9^*= 0.67 (SD 0.19); mean *Tie2^fl/fl/Cre-^*= 0.87 (SD 0.36); *p=* 0.21) (Fig 5B), contrasting significantly lower Tie2 protein expression in the *Tie2^ΔE9^* knockout mice. *Tie1* mRNA expression was significantly increased in arterioles (mean *Tie2^ΔE9^*= 1.38 (SD 0.26); mean *Tie2^fl/fl/Cre-^*= 0.86 (SD 0.35); *p=* 0.007), but not in glomeruli (mean *Tie2^ΔE9^*= 0.40 (SD 0.03); mean *Tie2^fl/fl/Cre-^*= 0.47 (SD 0.08); *p=* 0.06) of *Tie2^ΔE9^* knockout mice (Fig 5C). Analysis of gene expression in kidney microvascular compartments also revealed a significant, two-fold increase in *Ang1* expression in arterioles of *Tie2^ΔE9^* knockout mice (mean *Tie2^ΔE9^*= 0.107 (SD 0.045); mean *Tie2^fl/fl/Cre-^*= 0.059 (SD 0.035); *p=* 0.036), but not in glomeruli (mean *Tie2^ΔE9^*= 0.022 (SD 0.007); mean *Tie2^fl/fl/Cre-^*= 0.023 (SD 0.008); *p=* 0.70) (Fig 5D), while *Ang2* expression was unchanged in arterioles (mean *Tie2^ΔE9^*= 0.016 (SD 0.012); mean *Tie2^fl/fl/Cre-^*= 0.017 (SD 0.011); *p=* 0.80) and glomeruli (mean *Tie2^ΔE9^*= 0.054 (SD 0.004), mean *Tie2^fl/fl/Cre-^*= 0.056 (SD 0.016); *p=* 0.78) (Fig 5E).

**S4 table and S7 table**: we replaced NS by the *p* values.

**Comment Rev1-2:** Still no scale bar in the IHC panels (Fig 3).

**Response to Rev1-2:** We indeed did not add a scalebar in the IHC panels. Instead, we explicitly mentioned the magnification of each photomicrograph in figure legend 3A. We decided to take this approach as we were not able to add scale bars in a legible manner due to the large number and small size of the photomicrographs.

Based on this comment by the reviewer, we considered adding a scale bar to each photomicrograph, but by doing so we would falsify this figure. From our perspective, our initially chosen approach provides readers sufficient information to judge our results and to perform similar analyses in their own laboratory with our M&M and results as a reference. To allow readers to dive further into the details of our results, we propose to upload our Hamamatsu scans (NPDI-files, total size= 33GB) to a PLOS ONE repository, for everybody to access, provided PLOS ONE has such a repository available. A third party solution could be the freely available Zenodo database, provided by the CERN Data Platform to support open access data availability, that uniquely tags the scans by a DOI. We ask the editor for advice on this, and will adapt the manuscript accordingly. We will provide a short manual how to access the scans by use of Aperio ImageScope software.

We propose to add a sentence in the Methods sections to point to the DOI for NPDI-file access:

**Line 144**: Raw NPDI files are available via repository DOIxxx.

**Comment Rev1-3:** In Fig 2B Tie2 quantitation by ELISA is presented. However in the Methods section there is no information about reagents (kits) used in this experiment and about sensitivity range of the assay, which is important given that authors made conclusions based on quite low levels of protein (within 0-1 pg/ml range, unfortunately it is impossible to determine exact values from the graph bar and authors have not provided that information in Results section or Figure legend).

**Response to Rev1-3:** We agree with reviewer #1 that the information on the ELISA was concise. To determine Tie2 protein in tissue homogenates, an input of 100 – 500 ug total protein in the tissue homogenate was chosen. With this input, an OD value was obtained which was approximately in the middle of the calibration curve (125 – 8,000 pg/mL). OD values of the samples were calculated to Tie2 protein using the calibration curve (Tie2 pg/ml), corrected for volume (Tie2 pg) and normalized to protein input (Tie2 pg/ug).

The text has been adapted in the Methods section:

**Line 121-127**: Briefly, cryosections of organs were used to prepare tissue homogenates in RIPA buffer containing protease inhibitor, phosphatase inhibitor and activated Na_3_VO_4_. Total protein concentration was determined by *DC™* Protein Assay Kit II (#5000112; Bio-Rad Laboratories B.V., Veenendaal, The Netherlands). Tie2 protein expression was quantified by use of Mouse Tie2 Quantikine ELISA Kit (cat# MTE200, sensitivity 40.2 pg/ml and assay range 125.0 – 8,000 pg/ml) according to manufacturer’s instruction (R&D Systems, Minneapolis, MN, USA). Amounts of Tie2 protein were normalized for the total protein input of tissue homogenate and expressed as pg/µg of total protein.
